# The Associations Between Smoking Habits and Serum Triglyceride or Hemoglobin A1c Levels Differ According to Visceral Fat Accumulation

**DOI:** 10.2188/jea.JE20150086

**Published:** 2016-04-05

**Authors:** Michiko Koda, Itsuko Kitamura, Tomohiro Okura, Rei Otsuka, Fujiko Ando, Hiroshi Shimokata

**Affiliations:** 1 College of Bioscience and Biotechnology, Chubu University, Kasugai, Aichi, Japan; 1 中部大学応用生物学部; 2 Section of Longitudinal Study of Aging, National Institute for Longevity Sciences (NILS-LSA), National Center for Geriatrics and Gerontology, Obu, Aichi, Japan; 2 国立長寿医療研究センター; 3 Division of Liberal Arts and Sciences, Aichi Gakuin University, Nisshin, Aichi, Japan; 3 愛知学院大学教養部; 4 Faculty of Health and Sport Sciences, University of Tsukuba, Tsukuba, Ibaraki, Japan; 4 筑波大学体育専門学群; 5 Faculty of Health and Medical Sciences, Aichi Shukutoku University, Nagakute, Aichi, Japan; 5 愛知淑徳大学健康医療科学部; 6 Graduate School of Nutritional Sciences, Nagoya University of Arts and Sciences, Nisshin, Aichi, Japan; 6 名古屋学芸大学大学院栄養科学研究科

**Keywords:** smoking habits, visceral fat, interaction, serum triglycerides, hemoglobin A1c, 喫煙習慣, 内臓脂肪, 交互作用, 血清中性脂肪, ヘモグロビンA1c

## Abstract

**Background:**

Whether smokers and former smokers have worse lipid profiles or glucose levels than non-smokers remains unclear.

**Methods:**

The subjects were 1152 Japanese males aged 42 to 81 years. The subjects were divided according to their smoking habits (nonsmokers, former smokers, and current smokers) and their visceral fat area (VFA) (<100 cm^2^ and ≥100 cm^2^).

**Results:**

The serum triglyceride (TG) levels of 835 males were assessed. In the VFA ≥100 cm^2^ group, a significantly greater proportion of current smokers (47.3%) exhibited TG levels of ≥150 mg/dL compared with former smokers (36.4%) and non-smokers (18.8%). The difference in TG level distribution between former smokers and non-smokers was also significant. However, among the subjects with VFA of <100 cm^2^, the TG levels of the three smoking habit groups did not differ. The serum hemoglobin A1c (HbA1c) levels of 877 males were also assessed. In the VFA <100 cm^2^ group, significantly higher proportions of current smokers (17.9%) and former smokers (14.9%) demonstrated HbA1c levels of ≥5.6% compared with non-smokers (6.3%). In contrast, in the VFA ≥100 cm^2^ group, significantly fewer former smokers displayed HbA1c levels of ≥5.6% compared with non-smokers and current smokers. Furthermore, the interaction between smoking habits and VFA was associated with the subjects’ TG and HbA1c concentrations, and the associations of TG and HbA1c concentrations and smoking habits varied according to VFA.

**Conclusions:**

Both smoking habits and VFA exhibited associations with TG and HbA1c concentrations. The associations between smoking habits and these parameters differed according to VFA.

## 要約

**背景：:**

喫煙者や禁煙者は、脂質状態や血糖値に関して非喫煙者よりも好ましくない状態にあるかどうか依然として明確ではない。

**方法：:**

対象者は42-81歳の日本人男性1152名であった。対象者を喫煙習慣（非喫煙、禁煙、喫煙の3群）で分け、さらに内臓脂肪面積（VFA）（100cm^2^未満群と100cm^2^以上群）で分割した。

**結果：:**

血清中性脂肪（TG）値について835名で検討した。VFA100cm^2^ 以上では、喫煙群のTG150mg/dL以上の人の割合（47.3%）は、禁煙群（36.4%）や非喫煙群（18.8%）と比較して有意に高かった。禁煙群と非喫煙群間においてもTG値の分布は有意に異なっていた。しかし、VFA100 cm^2^未満では、3群間で違いは認められなかった。血清ヘモグロビンA1c（HbA1c）値に関しても877名で同様に検討した。VFA100 cm^2^未満では、喫煙群（17.9%）と禁煙群（14.9%）のHbA1c5.6%以上の人の割合は、非喫煙群（6.3%）と比較して有意に高かった。反対に、VFA100 cm^2^ 以上では、それらの割合は非喫煙群や喫煙群よりも禁煙群で有意に低かった。また、喫煙習慣とVFAとの交互作用はTG値およびHbA1c値でともにみられたが、TG値やHbA1c値と喫煙習慣との関係はVFAによって異なっていた。

**結論：:**

喫煙もVFAもそれぞれTG値やHbA1c値と関係することが認められた。喫煙習慣とそれらとの関係はVFAの大小によって異なっていた。

## INTRODUCTION

Smoking is a known risk factor for arteriosclerosis and diabetes mellitus (DM). Previous studies have reported that smokers have higher serum triglyceride (TG) and blood glucose concentrations and lower high-density lipoprotein cholesterol (HDL-C) concentrations than non-smokers.^1^^–^^6^ However, findings regarding whether smokers and former smokers have worse lipid profiles or glucose levels than non-smokers have been inconsistent. Freeman et al reported that smoking has little impact on TG levels in males.^3^ In addition, several studies have demonstrated that the insulin resistance and fasting serum glucose levels of current smokers and non-smokers do not differ significantly,^7^^–^^10^ and light smoking was found to reduce the risk of type 2 DM in lean males.^11^

Differences in weight or fat distribution might have been responsible for the inconsistent results obtained by previous studies. Body weight and abdominal visceral fat (VF) volume are strongly associated with higher serum TG concentrations,^12^^,^^13^ lower serum HDL-C concentrations,^14^ and insulin resistance.^15^^–^^17^ In addition, smoking and/or smoking cessation might affect body weight and fat distribution. For example, many studies have indicated that smokers tend to have lower body weights than non-smokers,^18^^–^^20^ and smoking cessation is known to be associated with substantial weight gain.^20^^–^^22^ It is also known that smoking is a risk factor for abdominal VF accumulation.^19^^,^^23^ Thus, the relationships among smoking, body weight, VF, serum lipids, and serum glucose levels are complex. Some previous studies have analyzed the associations of the interaction between smoking and body mass index (BMI) and lipid or glucose metabolism.^11^^,^^24^^,^^25^ However, BMI is not necessarily an accurate measure of VF accumulation, as it is influenced by both adipose and lean tissue.

Therefore, the purpose of this study was to investigate the interaction between smoking habits and VF area (VFA), which was measured using computerized tomography (CT) scans, and serum lipid (Study 1) or serum glucose (Study 2) concentrations.

## METHODS

### Subjects

**Figure 1.**
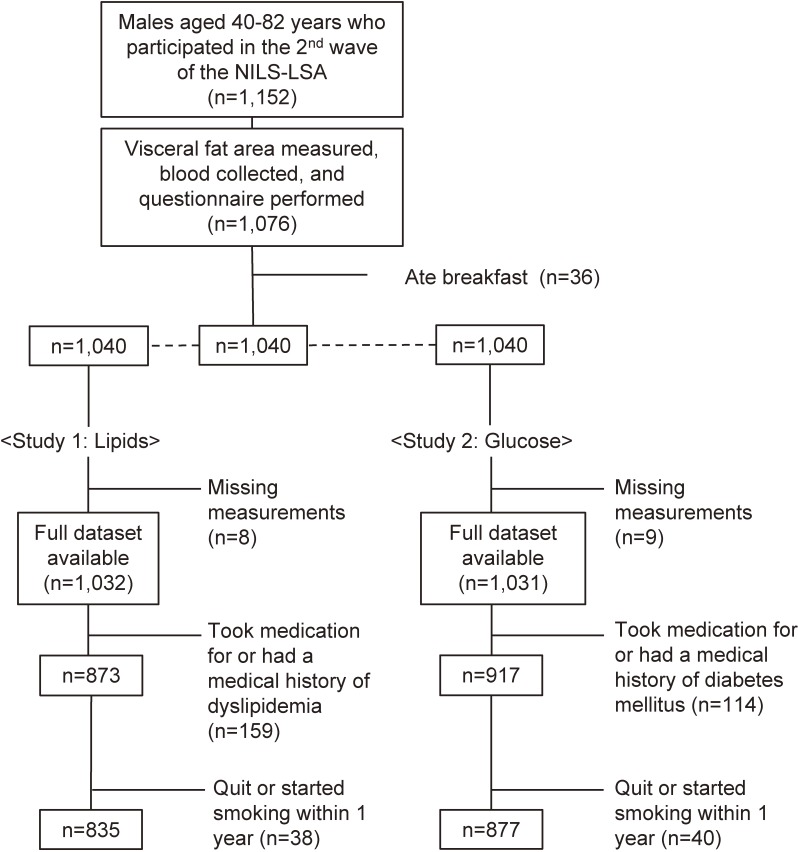
Sampling procedure.

The subjects were selected from among the 1152 Japanese males (age range, 40 to 82 years) who participated in the second wave of the National Institute for Longevity Sciences-Longitudinal Study of Aging (NIL-LSA; the second wave examinations were conducted from April 2000 to May 2002). The NIL-LSA involved gender- and age-stratified samples that were randomly selected from the local neighborhood surrounding our institute. The purpose of the present study was explained to each subject before written consent was obtained. The design of the NILS-LSA has been described elsewhere.^26^ Subjects who had eaten breakfast were excluded. The smoking data and medical history of the subjects, including any medication and/or treatment that they had received, were self-reported by the subjects using a questionnaire. Non-smokers were defined as people that had never smoked. Former smokers were defined as those who had quit smoking more than 1 year ago and had not smoked since, and current smokers were defined as those who had been smoking for more than 1 year. The subjects of Study 1 were 835 males (72% of the NIL-LSA cohort) who did not have a medical history of dyslipidemia and were not taking medication for the condition. The subjects of Study 2 were 877 males (76% of the NIL-LSA cohort) who did not have a medical history of DM and were not taking medication for the condition (Figure 1).

### Measurements

The subjects had their body weight (kg) assessed in the morning after overnight fasting. Body weight was measured using digital scales with the subjects only wearing underwear. BMI was calculated as weight (kg) divided by height (m) squared. VFA (cm^2^) and subcutaneous fat area (SFA; cm^2^) were measured using CT scans of the abdomen taken at the umbilical level (L4–L5). All CT scans (SCT-6800TX; Shimadzu, Osaka, Japan) were performed with the subjects in the supine position. VFA and SFA were calculated using computer software (Fat Scan; N2 Systems, Osaka, Japan)^27^ in accordance with a previously described procedure.^28^

Venous blood was collected in tubes containing ethylenediaminetetraacetic acid (EDTA, disodium salt, 50 mM) after overnight fasting, and then the samples’ serum total cholesterol (TC; mg/dL), HDL-C (mg/dL), TG (mg/dL), glucose (mg/dL), and hemoglobin A1c (HbA1c) (%) levels were analyzed. In this study, the HbA1c data are given in Japan Diabetes Society (JDS) units, which are calculated by subtracting 0.4 from standard HbA1c values.^29^

Dietary intake was estimated from 3-day food records, which were accompanied by before and after photos of each meal.^30^ Dietitians checked the food records while examining the photos. Physical activity was estimated from interviews regarding the subjects’ physical activity in the past year.^31^

### Data analysis

The subjects were divided into three groups (non-smokers, former smokers, and current smokers) based on the results of the smoking habits questionnaire. In addition, the subjects were also grouped into two VFA categories (VFA <100 cm^2^ and VFA ≥100 cm^2^), which were chosen based on the criterion for visceral obesity developed by the Japanese Society of Obesity.^32^ Comparisons of means among the smoking habit and VFA groups were performed using the Steel-Dwass test. Two-way ANOVA was used to evaluate the effects of the interaction between VFA categories and smoking habits on the subjects’ serum concentrations of TG, HDL-C, TC, glucose, and HbA1c. Prior to the analysis, the subjects’ HDL-C, TG, fasting glucose, and HbA1c concentrations were converted to natural logarithms (ln) to normalize their skewed distributions. Comparisons between frequencies were performed using chi-squared test. In Study 1, the following criteria were used to diagnose dyslipidemia: TC ≥220 mg/dL, HDL-C <40 mg/dL, and TG ≥150 mg/dL. These criteria were developed by the Japan Atherosclerosis Society.^33^ In Study 2, the following criteria were employed to diagnose metabolic syndrome: fasting glucose ≥110 mg/dL and HbA1c (JDS) ≥5.6%. The former criteria were developed by the Japan Society of Obesity,^32^ and the latter criteria were used as a substitute for fasting glucose in the National Health and Nutrition Examination Survey.^34^ Probability values of less than 0.05 were regarded as significant. The data were analyzed using the SAS statistical software package (release 9.3; SAS Institute, Cary, NC, USA).

### Ethics

This study was performed in accordance with the World Medical Association Declaration of Helsinki—Ethical Principles for Medical Research Involving Human Subjects. All of the procedures performed in the NILS-LSA were approved by the Committee of Ethics for Human Research at the National Center for Geriatrics and Gerontology. In the NILS-LSA study, an explanatory meeting was held 2 weeks before the start of the examinations.^26^ At the meeting, the purpose of the study and the procedures for each examination were fully explained to the subjects. All of the participants provided written informed consent. All data were analyzed collectively, and the subjects’ privacy was protected.

## RESULTS

### Study 1

The characteristics of subjects in Study 1 are shown in Table 1. Compared with non-smokers (22.8%) and current smokers (30.0%), a significantly greater proportion of former smokers (47.2%) exhibited VFA ≥100 cm^2^ (*P* < 0.01). In the VFA ≥100 cm^2^ group, non-smokers had the lowest mean serum TG concentration (*P* < 0.01), and that of former smokers was significantly lower than that of current smokers (*P* < 0.01). Among subjects with VFA <100 cm^2^, the serum TG levels of the three smoking habit groups did not differ, but current smokers had a significantly lower mean serum HDL-C concentration than former smokers (*P* < 0.05). In the VFA ≥100 cm^2^ group, the mean serum HDL-C concentration of current smokers was significantly lower than that of non-smokers (*P* < 0.01). The mean serum TC concentrations of the three smoking habit groups did not differ in either the VFA <100 cm^2^ or VFA ≥100 cm^2^ groups. Similarly, energy intake and physical activity did not differ among the three smoking habit groups in either the VFA <100 cm^2^ or VFA ≥100 cm^2^ group. Current smokers consumed more alcohol than the other groups.

**Table 1.** Characteristics of Study 1 subjects according to their smoking habits

	All *n* = 835	VFA <100 cm^2^			VFA ≥100 cm^2^		
	
		Non-smokers	Former smokers	Current smokers	Non-smokers	Former smokers	Current smokers
		*n* = 132	*n* = 193	*n* = 207	*n* = 69	*n* = 143	*n* = 91
Age, years	60.5 (10.8)	58.7 (11.1)	61.9 (11.2)**	58.7 (10.5)^††^	61.5 (9.4)	63.2 (10.4)	59.0 (10.3)^††^
BMI, kg/m^2^	22.9 (2.8)	21.9 (2.3)	21.9 (2.4)	21.3 (2.3)*^†^	25.1 (2.1)	25.0 (2.4)	24.8 (2.4)
VFA, cm^2^	90.2 (49.8)	57.9 (24.9)	61.9 (24.5)	59.9 (25.5)	137.8 (30.6)	144.4 (40.7)	144.4 (35.8)
Subcutaneous fat area, cm^2^	109.0 (47.7)	96.2 (38.6)	96.1 (39.9)	86.9 (40.6)*^†^	140.1 (49.8)	142.4 (44.4)	128.6 (46.3)^†^
Physical activity, METS*min/yr/10^3^	701 (876)	709 (88)	700 (90)	713 (98)	701 (71)	680 (70)	691 (82)
Total cholesterol, mg/dL	207.1 (32.7)	208.1 (33.0)	206.3 (34.6)	204.3 (32.3)	211.5 (33.9)	210.2 (29.9)	205.3 (32.1)
HDL cholesterol, mg/dL	57.9 (14.9)	61.7 (13.5)	62.4 (16.3)	58.7 (16.1)^†^	56.5 (13.3)	52.6 (11.0)*	50.8 (12.0)**
Triglycerides, mg/dL	120.6 (71.2)	110.9 (53.7)	101.8 (48.0)	112.0 (63.1)	111.4 (39.9)	143.3 (78.5)**	179.8 (109.3)**^††^

	*n* = 777	*n* = 125	*n* = 180	*n* = 190	*n* = 64	*n* = 135	*n* = 83

Energy intake, kcal	2301 (411)	2318 (414)	2308 (379)	2270 (408)	2288 (443)	2279 (387)	2379 (489)
Alcohol intake, g/day	16.5 (20.3)	15.9 (19.0)	15.9 (19.2)*	18.6 (23.0)**	12.6 (15.7)	14.3 (15.9)	27.8 (27.4)**^††^

BMI, body mass index; HDL, high-density lipoprotein; METS, metabolic equivalents; VFA, visceral fat area.

Data are shown as mean (standard deviation) values.

The differences between groups were analyzed using the Steel-Dwass test.

*P*-values: Compared with non-smokers: *P < 0.05, **P < 0.01; Compared with former smokers: ^†^*P* < 0.05, ^††^*P* < 0.01.

**Figure 2.**
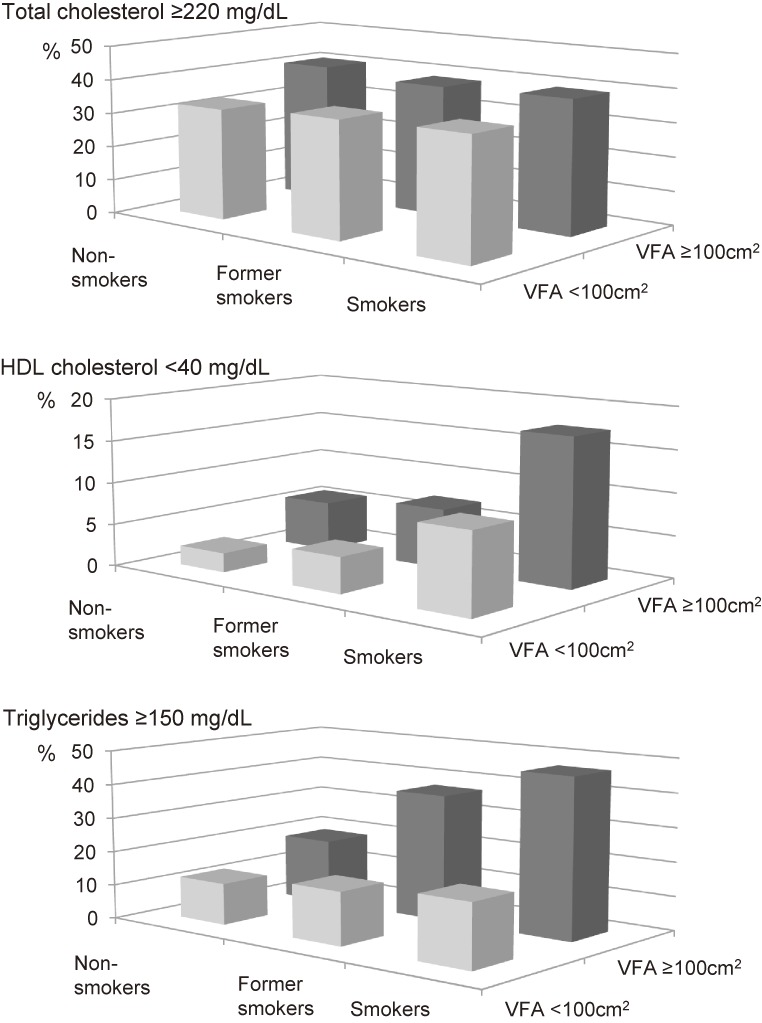
Frequencies of high serum total cholesterol and triglyceride levels and low serum HDL cholesterol concentrations.

Figure 2 shows the prevalence of elevated serum TC and TG levels and lower serum HDL-C concentrations. In the VFA ≥100 cm^2^ group, a significantly higher proportion of current smokers (47.3%) exhibited serum TG levels ≥150 mg/dL compared with former smokers (36.4%) and non-smokers (18.8%). In the VFA <100 cm^2^ group, there was no difference in the frequency of serum TG levels ≥150 mg/dL among the three smoking habit groups. The frequency of serum HDL-C levels <40 mg/dL was highest in current smokers in both the VFA <100 cm^2^ (current smokers: 9.7%, former smokers: 3.6%, non-smokers: 2.3%) and VFA ≥100 cm^2^ groups (current smokers: 17.6%, former smokers: 7.0%, non-smokers: 5.8%).

Two-way ANCOVA demonstrated that the interaction between VFA and smoking habits was significantly associated with the serum ln(TG) level but not serum ln(TC) or ln(HDL-C) levels (Table 2).

**Table 2.** Two-way factorial ANOVA

			F-value	*P*-value
Study 1	ln(Total cholesterol)	VFA	1.67	0.197
		smoking	1.21	0.299
		VFA*smoking	0.21	0.812
	ln(HDL cholesterol)	VFA	60.49	<0.001
		smoking	7.24	<0.001
		VFA*smoking	1.14	0.320
	ln(Triglycerides)	VFA	96.52	<0.001
		smoking	12.17	<0.001
		VFA*smoking	5.53	0.004

Study 2	ln(Glucose)	VFA	40.16	<0.001
		smoking	0.78	0.461
		VFA*smoking	5.75	0.003
	ln(HbA1c)	VFA	23.54	<0.001
		smoking	5.91	0.003
		VFA*smoking	4.67	0.010

ANOVA, analysis of variance; HDL, high-density lipoprotein; VFA, visceral fat area.

Two-way ANOVA was used to evaluate the effects of the interaction between VFA categories and smoking habits on the natural logarithms of the serum concentrations of total cholesterol, HDL cholesterol, triglycerides, glucose, and HbA1c.

### Study 2

The characteristics of subjects in Study 2 are shown in Table 3. Compared with non-smokers (33.3%) and current smokers (35.5), a significantly greater proportion of former smokers (43.9%) had VFA ≥100 cm^2^ (*P* < 0.05). Non-smokers exhibited a significantly lower mean serum HbA1c level than current smokers (*P* < 0.01) among the subjects with VFA <100 cm^2^, whereas in the VFA ≥100 cm^2^ group, former smokers displayed a significantly lower mean HbA1c level than current smokers (*P* < 0.05). The mean serum glucose level was not associated with smoking habits in either the VFA <100 cm^2^ or VFA ≥100 cm^2^ groups. Energy intake did not differ among the three smoking habit groups in the VFA <100 cm^2^ or VFA ≥100 cm^2^ group. Current smokers consumed more alcohol than the other groups.

**Table 3.** Characteristics of Study 2 subjects according to their smoking habits

	All *n* = 877	VFA <100 cm^2^			VFA ≥100 cm^2^		
	
		Non-smokers	Former smokers	Current smokers	Non-smokers	Former smokers	Current smokers
		*n* = 142	*n* = 202	*n* = 196	*n* = 71	*n* = 158	*n* = 108
Age, years	60.3 (10.7)	58.8 (10.9)	61.2 (11.2)*	58.7 (10.7)^†^	61.9 (9.9)	62.9 (10.1)	59.0 (10.3)^††^
BMI, kg/m^2^	23.0 (2.8)	22.0 (2.2)	22.1 (2.3)	21.3 (2.4)*^††^	25.2 (2.1)	25.0 (2.3)	25.0 (2.3)
VFA, cm^2^	91.4 (49.7)	57.3 (24.0)	63.3 (24.1)*	58.4 (25.4)^†^	135.8 (29.2)	143.6 (39.3)	143.0 (36.6)
Subcutaneous fat area, cm^2^	111.5 (47.7)	95.1 (36.4)	99.2 (40.0)	87.5 (40.7)^††^	141.4 (50.8)	143.5 (43.8)	132.7 (45.7)
Physical activity, METS*min/yr/10^3^	701 (86)	712 (89)	699 (85)	716 (99)	706 (84)	681 (68)*	688 (80)
Glucose, mg/dL	100.3 (12.9)	96.8 (10.4)	100.0 (11.3)**	97.5 (11.1)^†^	106.8 (21.4)	102.2 (10.9)*	103.4 (14.2)
HbA1c, %	5.22 (0.49)	5.05 (0.40)	5.17 (0.38)*	5.23 (0.46)**	5.40 (0.81)	5.23 (0.38)*	5.39 (0.59)^†^

	*n* = 816	*n* = 134	*n* = 190	*n* = 179	*n* = 65	*n* = 150	*n* = 98

Energy intake, kcal	2303 (401)	2297 (411)	2309 (371)	2298 (392)	2279 (432)	2282 (366)	2359 (485)
Alcohol intake, g/day	16.1 (19.7)	10.7 (14.3)	15.7 (17.9)*	17.9 (23.2)**	12.3 (15.5)	14.6 (15.8)	25.4 (26.2)**^††^

BMI, body mass index; METS, metabolic equivalents; VFA, visceral fat area.

Data are shown as mean (standard deviation) values.

The differences between groups were analyzed using the Steel-Dwass test.

*P*-values: Compared with non-smokers: *P < 0.05, **P < 0.01; Compared with former smokers: ^†^*P* < 0.05, ^††^*P* < 0.01.

**Figure 3.**
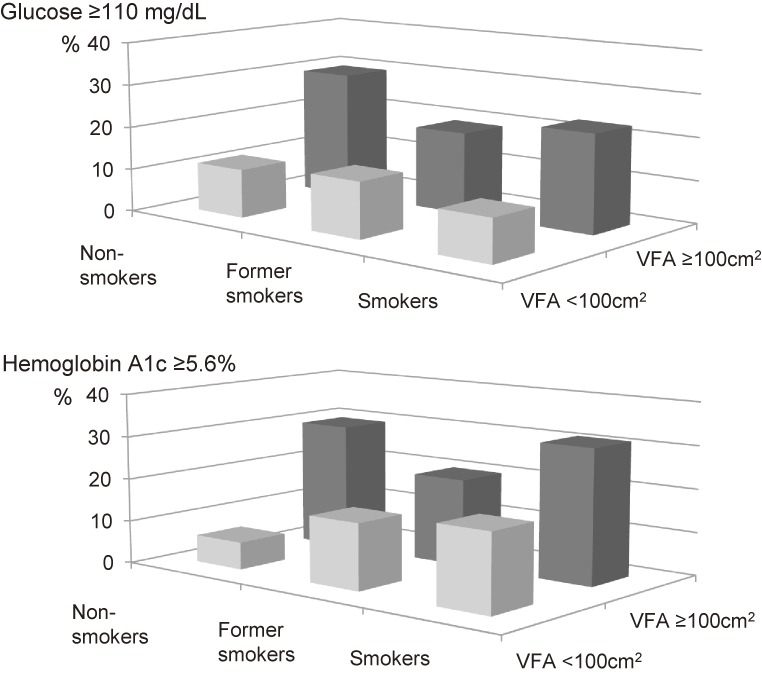
Frequencies of high serum glucose and hemoglobin A1c concentrations.

Figure 3 shows the prevalence of elevated glucose and HbA1c concentrations. In the VFA <100 cm^2^ group, serum HbA1c levels of ≥5.6% were detected in significantly higher proportions of current smokers (17.9%) and former smokers (14.9%) than non-smokers (6.3%), whereas in the VFA ≥100 cm^2^ group, serum HbA1c levels of ≥5.6% were observed in significantly greater proportions of current smokers (31.5%) and non-smokers (29.6%) than former smokers (19.6%).

The relationships of the interaction between VFA and smoking habits and the subjects’ serum glucose and HbA1c concentrations are shown in Table 3. Two-way ANCOVA demonstrated that the interaction between VFA and smoking habits was significantly associated with the subjects’ serum ln(glucose) (*P* = 0.003) and ln(HbA1c) levels (*P* = 0.010). However, no relationship was detected between the subjects’ smoking habits and their serum glucose levels.

## DISCUSSION

Our data showed that both smoking and VF accumulation were associated with serum TG, HDL-C, and HbA1c concentrations, and that the interaction between smoking habits and VFA was also associated with serum TG and HbA1c levels but not serum HDL-C concentrations. The former two parameters demonstrated different relationships with the interaction between smoking habits and VFA, and the associations between smoking habits and these parameters differed according to VFA. The findings of the present study suggest that smoking is only weakly associated with the serum TG concentrations among men with little VF. However, in men with large amounts of VF, the relationship between smoking and serum TG concentrations might be stronger. Although smoking might be associated with HbA1c concentrations in men with little VF, it might not be as strongly related to HbA1c concentrations as VFA. Moreover, while the serum HDL-C level might be related to VFA, it may also be associated with smoking habits, irrespective of the amount of VF present.

These results suggest that differences in VF accumulation might have been responsible for the inconsistent results obtained by previous studies regarding the relationships between smoking and serum TG or HbA1c levels. Freeman et al^3^ reported that smoking had little impact on TG. However, the subjects of this study had a mean BMI of around 25 kg/m^2^, so they probably did not have much VF. Some studies have found that the relationship between smoking and serum fasting glucose concentrations is stronger in lean men than in obese men.^24^^,^^35^^,^^36^ Our data support these findings. Henkin et al^9^ reported that there was no association between smoking and insulin sensitivity. The mean BMI of their participants was 29.4 (standard deviation, 5.9) kg/m^2^; thus, it is presumed that most of the participants in their study were overweight or obese.

The mechanism responsible for the interaction between smoking habits and VF accumulation is poorly understood, although it seems to involve lipoprotein lipase (LPL) activity and certain adipocytokines. For example, the pre-heparin plasma LPL mass was found to be negatively correlated with TG concentration and insulin resistance and positively correlated with HDL-C concentration.^37^ Increased serum levels of adiponectin (an adipocytokine) have been found to be associated with higher HDL-C levels and lower TG^38^ and glucose concentrations.^39^

Abdominal VFA itself is strongly correlated with higher TG and lower HDL-C levels, as well as with insulin resistance.^12^^,^^15^^–^^17^ VF might release free fatty acids into the portal circulation, causing them to enter the liver directly, and excess free fatty acids might enhance hepatic TG synthesis and insulin resistance.^38^^,^^40^

However, smokers exhibit reduced plasma post-heparin LPL activity and adiponectin levels,^41^^–^^43^ and both of these parameters were found to be inversely correlated with VFA.^37^ We did not measure the serum levels of these substances, so further investigation of these relationships is required.

In the present study, about 35% of the subjects were smokers, which was similar to the prevalence obtained in the 2011 National Health and Nutrition Examination Survey (32.4%).^34^

Energy balance is associated with body weight and VF accumulation, and physical activity improves serum lipid levels and insulin resistance.^44^^,^^45^ However, neither of these parameters differed among the three smoking habit groups in the present study. Smokers consumed more alcohol than non-smokers in both the VFA <100 cm^2^ and VFA ≥100 cm^2^ groups in the present study, and similar findings were obtained in previous studies.^36^^,^^46^^,^^47^ Alcohol consumption is also positively associated with serum HDL-C levels in both smokers and non-smokers,^48^^,^^49^ and excessive alcohol consumption is associated with higher serum TG concentrations.^48^^,^^50^ In the present study, we found that controlling for alcohol intake had little effect on our results (data not shown).

The current study had some limitations. First, we did not examine the number of cigarettes smoked per day or the duration since smoking cessation in our analysis, and the effects of smoking on serum lipid levels and DM^2^^,^^11^ have been shown to be dose-dependent. The number of years since smoking cessation has also been shown to be associated with the risk of DM.^51^^–^^53^ Thus, a longitudinal analysis examining the effects of the interaction between smoking and VF accumulation on serum lipid and glucose levels needs to be performed in the future. Second, passive smokers were not distinguished from non-smokers. It has been reported that passive smoking is associated with an increased risk of coronary heart disease^54^^–^^56^ and type 2 DM,^57^ and the non-smoker group in the present study might have included some passive smokers.

In conclusion, smoking was found to be associated with higher serum TG, glucose, and HbA1c levels and lower serum HDL-C levels. The associations between smoking habits and these parameters differed according to VFA. However, the interaction between VFA and smoking habits exhibited different relationships with the serum levels of TG and HbA1c; VFA had a stronger association with serum HbA1c level than smoking, and, among subjects with a lot of VF, elevated serum TG levels were detected in significantly greater proportions of smokers and former smokers than non-smokers. These findings suggest that not smoking and not accumulating VF are both important for health. In particular, middle-aged and elderly men with a lot of VF should not smoke or should quit smoking because smoking exhibits a strong adverse association with serum TG concentration.

## ONLINE ONLY MATERIAL

Abstract in Japanese.
