# Supplementary material for: The Associations Between Smoking Habits and Serum Triglyceride or Hemoglobin A1c Levels Differ According to Visceral Fat Accumulation
Source: J Epidemiol. 2016 Apr 5;26(4):208–15. doi: 10.2188/jea.JE20150086 (PMC4808688; doi:10.2188/jea.JE20150086)
Supplement: Abstract in Japanese. [file je-26-208-s001.pdf]

喫煙と血清中性脂肪、ヘモグロビンA1cとの関係は内臓脂肪によって異なる

甲田道子[1,2]、北村伊都子[2,3]、大藏倫博[2,4]、大塚礼[2]、安藤富士子[2,5]、下方浩史[2,6]

1. 中部大学応用生物学部
2. 国立長寿医療研究センター
3. 愛知学院大学教養部
4. 筑波大学体育専門学群
5. 愛知淑徳大学健康医療科学部
6. 名古屋学芸大学大学院栄養科学研究科

要約

背景：喫煙者や禁煙者は、脂質状態や血糖値に関して非喫煙者よりも好ましくない状態にあるかどうか依然として明確ではない。

方法：対象者は42-81歳の日本人男性1152名であった。対象者を喫煙習慣（非喫煙、禁煙、喫煙の3群）で分け、さらに内臓脂肪面積（VFA）（100cm^{2}未満群と100cm^{2}以上群）で分割した。

結果：血清中性脂肪（TG）値について835名で検討した。VFA100cm^{2}以上では、喫煙群のTG150mg/dL以上の人の割合（47.3%）は、禁煙群（36.4%）や非喫煙群（18.8%）と比較して有意に高かった。禁煙群と非喫煙群間においてもTG値の分布は有意に異なっていた。しかし、VFA100 cm^{2}未満では、3群間で違いは認められなかった。血清ヘモグロビンA1c（HbA1c）値に関しても877名で同様に検討した。VFA100 cm^{2}未満では、喫煙群（17.9%）と禁煙群（14.9%）のHbA1c5.6%以上の人の割合は、非喫煙群（6.3%）と比較して有意に高かった。反対に、VFA100 cm^2 以上では、それらの割合は非喫煙群や喫煙群よりも禁煙群で有意に低かった。また、喫煙習慣とVFAとの交互作用はTG値およびHbA1c値でともにみられたが、TG値やHbA1c値と喫煙習慣との関係はVFAによって異なっていた。

結論：喫煙もVFAもそれぞれTG値やHbA1c値と関係することが認められた。喫煙習慣とそれらとの関係はVFAの大小によって異なっていた。

キーワード

喫煙習慣、内臓脂肪、交互作用、血清中性脂肪、ヘモグロビンA1c
